# Modified ERAS protocol using preoperative oral rehydration therapy: outcomes and issues

**DOI:** 10.1007/s00540-013-1769-3

**Published:** 2014-01-03

**Authors:** Hideki Taniguchi, Toshio Sasaki, Hisae Fujita, Hiroko Kobayashi, Rieko Kawasaki, Minako Goloubev, Tomomi Ishikawa, Osami Takano, Takashi Ogata, Haruhiko Cho, Takaki Yoshikawa

**Affiliations:** 1 School of Nutrition and Dietetics, Kanagawa University of Human Services, 1-10-1 Heisei, Yokosuka, Kanagawa 238-8522 Japan; 2 Department of Anesthesiology, Kanagawa Cancer Center, 2-3-2 Nakao, Asahi-ku, Yokoahama, Kanagawa 241-8515 Japan; 3 Department of Gastrointestinal Surgery, Kanagawa Cancer Center, 2-3-2 Nakao, Asahi-ku, Yokohama, Kanagawa 241-8515 Japan

## Introduction



In recent years, the use of postoperative recovery management protocols such as the “enhanced recovery after surgery (ERAS^®^) protocol” [1] and “fast track program” [2] is steadily spreading to clinical institutions across the country. These protocols involve evidence-based interventions to enhance patient recovery after surgery, which can be realized by a multidisciplinary team approach performed during perioperative periods. As the outcomes of the protocols, shorter hospitalization, reduced perioperative complications, and medical cost reduction are anticipated, and consistent outcomes are actually obtained [3]. At the same time, since these protocols have their origins in Europe and America, there are some aspects which are not consistent with Japanese medical culture and situation. These are (1) patients are very reluctant to accept shorter hospitalization (though length of hospital stay following operative procedures is taken as one of the endpoints in the protocol), (2) patient education and counseling are still not sufficient to adequately perform early postoperative mobilization and oral intake in patients, and more nurses and staff are required for such education and consulting services.

At our hospital, we practice a “modified ERAS^®^ protocol;” an enhanced recovery after surgery protocol modified to match medical practices and situations in this country [4]. In particular, preoperative fluid management using “preoperative oral rehydration therapy (PO-ORT)” is one of the important features, and we herein report the safety and efficacy of the “modified ERAS^®^ protocol”, including its outcomes and issues [4, 5].

## Modified ERAS^®^ protocol (MEP) employed in our hospital

The modified ERAS^®^ protocol (MEP) in our hospital is based on the basic principles of the ESSENSE project (essential strategy for early normalization after surgery with patient’s excellent satisfaction project) sponsored by the Japanese Society for Surgical Metabolism and Nutrition [6]. The principles encourage the following aims for earlier postoperative recovery of patients: (1) relieving surgery-related stress, (2) mobilizing patients as soon as possible, (3) nutrient intake by patients as soon as possible, and (4) relieving perioperative anxiety and increasing motivation to recover.

In our hospital, MEP is practiced for various types of surgery other than lower gastrointestinal surgery. Table 1 shows one of the MEP protocols used for gastric surgery in our hospital [4]. In addition to achieving the basic principles of the ESSENSE project, the MEP also aims to increase patient satisfaction and reduce the workload of nursing staff. Specifically, aggressive mobilization and oral intake, though recommended on the day of surgery in the ERAS^®^ protocol and fast track program, are practiced from the day after surgery in the MEP, because they are very likely to be a burden to both patients and nursing staff if initiated on the day of surgery [1, 2].

**Table 1:** Modified ERAS^®^ protocol (MEP) in gastric surgery

Operative day	−1	0	+1	+2	+3	+4	+5	+6	+7
Oral intake	Normal diet until midnight	Oral hydration solution (OS-1^®^) 3 h before surgery		Drink water and oral nutrition supplement (Ensure Liquid)	Liquid diet (3 steps up to soft diet every 2 days)				
Bowel preparation	1 g magnesium oxide and New Lecicarbon^®^ suppository								
		Combination of epidural analgesia (TH7-11) and general anesthesia during surgery							
Anesthesia and Analgesics		Continuous thoracic epidural infusion of analgesics after surgery	→	Removing epidural catheter					
		Nonsteroidal anti-inflammatory drug intravenously after surgery twice daily	→	Acetoaminophen three times a day orally	→	→	→		
Drain and NGT		No drain in distal gastrectomy, one or two drains in total gastrectomy NGT was removed immediately after surgery		Removing drain(s)					
ADL			Encourage to sit out of bed for more than 6 h	Encourage to walk the length of the ward	→	→	→	→	→
Antithrombopro phylaxis			None	Subcutaneous injection of antithrombotic agent (enoxaparin sodium or fondaparinux)	→	→	None	→	→
X-ray and blood exam.		○	○						○ (Check discharge criteria)

Note that interventions such as shortening the fasting period, not administering infusion solutions preoperatively, decreasing the use of laxatives, using epidural anesthesia, administering regular analgesics, not using postoperative gastric tubing, and preventing the formation of thrombi and emboli are all the same as those in the standard protocols. Other features of MEP at our hospital are the use of PO-ORT and administration of high-dose remifentanil. The outcomes obtained in the conduct of the MEP have been reported elsewhere as evidence.

### Preoperative oral rehydration therapy (PO-ORT)

It is reported in the ERAS^®^ protocol that postoperative insulin resistance can be reduced by taking hyper-concentrated carbohydrate (H-CHO loading), leading to an increase in postoperative recovery ability [1]. However, the products which have similar evidence of safety and efficacy as the H-CHO product recommended by the ERAS^®^ protocol (Table 2) are not commercially available in this country. Instead, “preoperative oral rehydration therapy (PO-ORT)” using an oral rehydration solution (ORS), classified as a carbohydrate-containing drink and generally called “clear” fluids, has been introduced in many hospitals in Japan (Table 2), and a shortened hospital stay and discontinuation of parenteral treatment in preoperative periods are commonly realized in these hospitals [5].

**Table 2:** Compositions of oral rehydration solution (ORS) and concentrated carbohydrate solution (H-CHO)

Product	Unit	ORS	H-CHO
		OS-f^®^	preOP^®^
Carbohydrate	%	2.5 (glucose 1.8)	12.6 (glucose 2.1)
Na^+^	mEq/l	50	22
K^+^	mEq/l	20	31
Mg^2^+	mEq/l	2.0	0.0
Lactate^−^	mEq/l	31	–
Cl^−^	mEq/l	50	0.2
P	mmol/l	2.0	0.0
Osmolarity	mOsm/l	Approx. 270	Approx. 240
pH	–	3.9	4.9

*ORS* oral rehydration solution, *H-CHO* hyper-concentrated carbohydrate drink

We investigated the occurrence of intravenous infusion-related events in the 1-year periods before and after introduction of PO-ORT (i.e., we compared July 2006–July 2007 with July 2007–July 2008). Using PO-ORT for preoperative fluid management of surgical patients, the occurrence rate of such events noticeably decreased to reflect the effect of not using intravenous solutions (27 ± 9 vs 15 ± 9 events/month, *P* < 0.01). The workload of ward nurses was also reduced by using PO-ORT [7].

PO-ORT is mentioned in the guideline published by the European Society of Anaesthesiology (“Perioperative fasting in adults and children”) in 2011. PO-ORT is as effective as intravenous infusion in terms of provision of water and electrolytes, and can reduce thirst, hunger, anxiety, and physical restriction due to intravenous infusion. Also, it can allow for safe introduction of general anesthesia in surgical patients without increasing the volume of gastric fluid [8]. Although ORS is recommended as the carbohydrate-containing drink in the guideline, its effect on insulin resistance is not addressed. Yatabe et al. investigated insulin resistance in healthy subjects using the glucose clamp technique. Since the carbohydrate concentration of ORS used in the study is as low as 2.5 % compared to H-CHO, its effect on insulin resistance is low, reflecting the concentration of carbohydrate. However, the results indicate that consumption of ORS may attenuate insulin resistance [9]. To position PO-ORT as one of the essential tools for postoperative enhanced recovery, its effect on enhancing postoperative recovery must be further studied.

### Intraoperative use of high-dose remifentanil

In our hospital, adequate maintenance of body fluids and electrolytes before surgery becomes possible with the use of MEP, which enables the use of sufficient doses of analgesics and sedatives during surgery [5]. Specifically, epidural analgesia with adequate local anesthetics and high doses of remifentanil are useful. With regard to the ERAS^®^ protocol and fast track program, there are a number of studies reporting on the effectiveness of epidural analgesia for accelerating postoperative recovery [1, 2], whereas there is very little discussion regarding the type and amount of intraoperative analgesics. In the ERAS^®^ protocols published in 2012 for elective colonic surgery, elective rectal/pelvic surgery, and pancreaticoduodenectomy, the level of evidence for intraoperative use of remifentanil is low, but the grade of recommendation is high. Actually, there are not many reports on intraoperative remifentanil [10–12].

We investigated the effect of intraoperative use of high-dose remifentanil on insulin resistance and muscle protein catabolism. Thirty-seven patients undergoing elective gastrectomy were randomly assigned to 2 groups receiving remifentanil at infusion rates of 0.1 μg/kg/min (group L: 18 patients) and 0.5 μg/kg/min (group H: 19 patients). Using a homeostasis model assessment as an index of insulin resistance (HOMA-IR), values varied within normal limits in both groups during surgery, exceeding normal limits at 12 h after surgery in both groups and being significantly elevated in group L. There were no significant differences in the 3-MH/Cr values between the 2 groups at any time point. The stress hormones (adrenocorticotropic hormone, cortisol, and adrenaline) were significantly elevated in group L at 60 min after the start of surgery and at the initiation of skin closure. There were no significant differences in insulin values, but blood glucose was significantly elevated in group L at 60 min after the start of surgery and at the start of skin closure. The results of this investigation strongly suggest that the use of high-dose remifentanil as an intraoperative analgesia for elective gastrectomy can help attenuate postoperative insulin resistance, although it may not reduce postoperative muscle protein catabolism [13]. Taken together, intraoperative use of high-dose remifentanil may be used effectively as one of the interventions for attenuating postoperative insulin resistance in addition to other interventions such as preoperative intake of carbohydrate-containing drinks, use of epidural analgesia during intra- and postoperative periods, and early mobilization of patients. However, further studies are needed to confirm whether it can enhance postoperative recovery.

## Outcomes and issues regarding MEP

### Outcomes

For patients undergoing elective gastrectomy, we performed a retrospective study to investigate postoperative recovery with respect to each endpoint. The control group (100 patients) received conventional preoperative management, and the MEP group (91 patients) received so-called MEP. As a result, patients in the MEP group started postoperative oral intake smoothly, and the amount of intake was relatively large. Reduced frequency of analgesic use was observed in the MEP group, and reduction ratio of postoperative body weight was also small. Although the ERAS^®^ protocol is usually studied in patients with lower digestive tract disease, many studies are now reporting on patients with upper digestive tract disease. For the outcomes, shortened hospital stay and reduction of patient readmission are reported as endpoints of enhanced recovery in laparoscopic elective gastrectomy [14].

PO-ORT is used at our hospital instead of H-CHO loading. In the ERAS^®^ protocol, it is recommended that “normal fluid and electrolyte balance” be maintained for patients at the time of entering an operating room to enhance their postoperative recovery [15].

As mentioned earlier, PO-ORT is as effective as intravenous infusion in terms of provision of water and electrolytes, and is a safe modality for preoperative body fluid management because it does not increase the volume of gastric fluid for introduction of general anesthesia. PO-ORT also helps reduce thirst, hunger, anxiety, and physical restriction due to intravenous infusion, providing a stress-free, comfortable environment for patients [5, 16]. Also, we investigated the change in preoperative body weight and amount of body water in patients who underwent elective gastrectomy. The body weight and body water were determined using a multi-frequency impedance method. Patients in the control group (50 patients) were managed with conventional preoperative management, and those in the MEP group (50 patients) were managed with so-called MEP. Body weight and body fluid both decreased more in the control group than in the MEP group. In the MEP group, it was possible to maintain “normal fluid and electrolyte balance” of preoperative body fluids by practicing PO-ORT in addition to preoperative measures such as shortened fasting time and reduction of laxative medication [17].

Furthermore, the effect on intraoperative anesthetic management was retrospectively investigated in the two groups, and it was found that urine volume significantly increased in the MEP group compared to the control group, even though there were no differences in the volume of bleeding, dose of intraoperative infusion, and use of vasopressors. These results suggest that doses of intraoperative infusion solutions may be reduced by preoperative management using MEP (Table 3).

**Table 3:** Effects of conventional preoperative patient management (control) and modified ERAS^®^ protocol (MEP) on intraoperative anesthetic management in patients for elective gastrectomy

	Control group	MEP group	*P* value
Sex (men/women)	15/35	21/29	*P* = 0.21
Age (years)	66 ± 10	62 ± 12	0.07
Anesthetic method (inhalation/intravenous)	18/32	13/37	0.28
Type of surgery (laparotomy/endoscopy)	22/28	21/29	0.16
Epidural anesthesia (no. of patients)	50	50	
BMI (kg/cm^2^)	22.1 ± 6.9	23.0 ± 6.0	0.09
ASAPS (I/II)	21/29	20/30	0.84
Vomiting due to anesthesia (no. of patients)	0	0	
Surgical time (min)	169 ± 61	188 ± 39	0.07
Bleeding (ml)	149 ± 195	171 ± 146	0.53
Volume of infusion (ml/kg/h)	9.1 ± 2.9	8.4 ± 2.5	0.18
Urine volume (ml/kg/h)	0.9 ± 0.6	1.3 ± 0.8	0.03
Use of vasopressor (times)	0.5 ± 0.6	0.4 ± 0.4	0.24

Statistical analysis: age and BMI were analyzed by unpaired *t*-test (significance level with two-sided, *P* = 0.05), and sex, ASAPS, and type of surgery were analyzed by chi-square test (significance level with two-sided, *P* = 0.05)

Values are mean ± SD

*ASAPS* American Society of Anesthesiologists physical status

### Issues

With regard to MEP, favorable effects are obtained in some variables such as safety, efficacy, and competence of team medicine. In particular, MEP using PO-ORT is not only an alternative tool for preoperative fluid management, but also has various effects such as increasing patient satisfaction and improving nursing services.

However, results on any of the variables in MEP are still not enough to allow for consistent evaluation of postoperative enhanced recovery. In terms of the effects on endpoints such as shortened hospitalization and reduction of medical cost (which are employed in the ERAS^®^ protocol initiated in Europe), it is too soon to say whether MEP practiced at our hospital does affect such endpoints.

This may be, as a whole, due to the fact that MEP used in our hospital does not seriously consider the length of hospitalization and reduction of medical cost as endpoints. Generally, patient satisfaction, reduction of workload of nurses, improvement of perioperative safety management, and encouragement of team medicine and a team approach are overwhelmingly important factors emphasized in this country. So, selection of endpoints pertinent to our medical surroundings and those which meet patient needs seem to be inevitable issues for MEP performed in this country as well as in our hospital.

## Conclusions

We have herein described a “modified ERAS^®^ protocol” (MEP) practiced in our hospital, including outcomes and issues. Preoperative oral rehydration therapy (PO-ORT) is one of the preoperative patient management modalities, based on evidence obtained in this country. MEP is practiced in our hospital as one of the specific approaches for the ESSENSE project (essential strategy for early normalization after surgery with patient’s excellent satisfaction project), sponsored by the Japanese Society for Surgical Metabolism and Nutrition. We believe that further studies may provide more evidence for MEP.
